# Experimentally obtained and computer-simulated X-ray asymmetric eight-beam pinhole topographs for a silicon crystal

**DOI:** 10.1107/S2053273319001499

**Published:** 2019-04-30

**Authors:** Kouhei Okitsu, Yasuhiko Imai, Yoshitaka Yoda, Yoshinori Ueji

**Affiliations:** a Nano-Engineering Research Center, Institute of Engineering Innovation, Graduate School of Engineering, The University of Tokyo, 2-11-16 Yayoi, Bunkyo-ku, Tokyo 113-8656, Japan; b Japan Synchrotron Radiation Research Institute, SPring-8, 1-1-1 Kouto, Mikazuki-cho, Sayo-gun, Hyogo 679-5198, Japan; c Rigaku Corporation, 3-9-12 Matsubara, Akishima-shi, Tokyo 196-8666, Japan

**Keywords:** X-ray diffraction, dynamical theory, multiple reflection, computer simulation, *n*-beam reflection, phase problem, silicon, protein crystallography

## Abstract

In this study, experimentally obtained eight-beam pinhole topographs for a silicon crystal using synchrotron X-rays were compared with computer-simulated images, and were found to be in good agreement. The experiment was performed with an asymmetric all-Laue geometry. However, the X-rays exited from both the bottom and side surfaces of the crystal. The simulations were performed using two different approaches: one was the integration of the *n*-beam Takagi–Taupin equation, and the second was the fast Fourier transformation of the X-ray amplitudes obtained by solving the eigenvalue problem of the *n*-beam Ewald–Laue theory as reported by Kohn & Khikhlukha [*Acta Cryst*. (2016 ▸), A**72**, 349–356] and Kohn [*Acta Cryst.* (2017 ▸), A**73**, 30–38].

## Abstract 2

Experimentally obtained eight-beam pinhole topographs for a silicon crystal were compared with computer simulations based on the *n*-beam Takagi–Taupin equation and Ewald–Laue theory.

## 1. Introduction   

We previously reported a derivation of the *n*-beam Takagi–Taupin (T-T) equation and an algorithm to integrate it (Okitsu, 2003 ▸; Okitsu *et al.*, 2006 ▸). We verified these by comparing computer-simulated and experimentally obtained topographs using a six-beam case (Okitsu *et al.*, 2003 ▸, 2006 ▸, 2011 ▸) and three-, four-, five-, six-, eight- and 12-beam cases (Okitsu *et al.*, 2012 ▸). Hereafter, Okitsu *et al.* (2006 ▸), Okitsu *et al.* (2011 ▸) and Okitsu *et al.* (2012 ▸) are denoted by O *et al.* 2006, O *et al.* 2011 and OIY 2012, respectively.

In OIY 2012, the *n*-beam T-T equation was derived from the *n*-beam Ewald–Laue (E-L) theory, and vice versa by their Fourier transformation, which explicitly revealed a simple relationship between them described by a Fourier transform. Ishiwata *et al.* (2010 ▸) reported X-ray rocking curves that were obtained by fast Fourier transformation of the X-ray amplitude in a three-beam topograph, and compared them with those computed by solving the eigenvalue problem of the three-beam E-L theory. In contrast, Heyroth *et al.* (2001 ▸) reported X-ray three-beam topographs experimentally obtained and computer simulated by coherently superposing the X-ray amplitude calculated based on the E-L theory.

Recently, Kohn & Khikhlukha (2016 ▸) and Kohn (2017 ▸) reported computer-simulated *n*-beam topographs (*n* = 6) obtained by fast Fourier transformation of the rocking amplitude calculated using the *n*-beam E-L theory. The present article reports the efficiency of Kohn’s method by comparing computer-simulated eight-beam pinhole topographs (E-L&FFT simulation) with the experimentally obtained and computer-simulated ones based on the *n*-beam T-T equation (T-T simulation) published in OIY 2012 {Figs. 5 [*S*_*x*_(T-T)], 5 [*E*_*x*_], 6 [*S*_*x*_(T-T)] and 6 [*E*_*x*_] (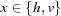)}.

## 2. Experimental   

**Figure 1:**
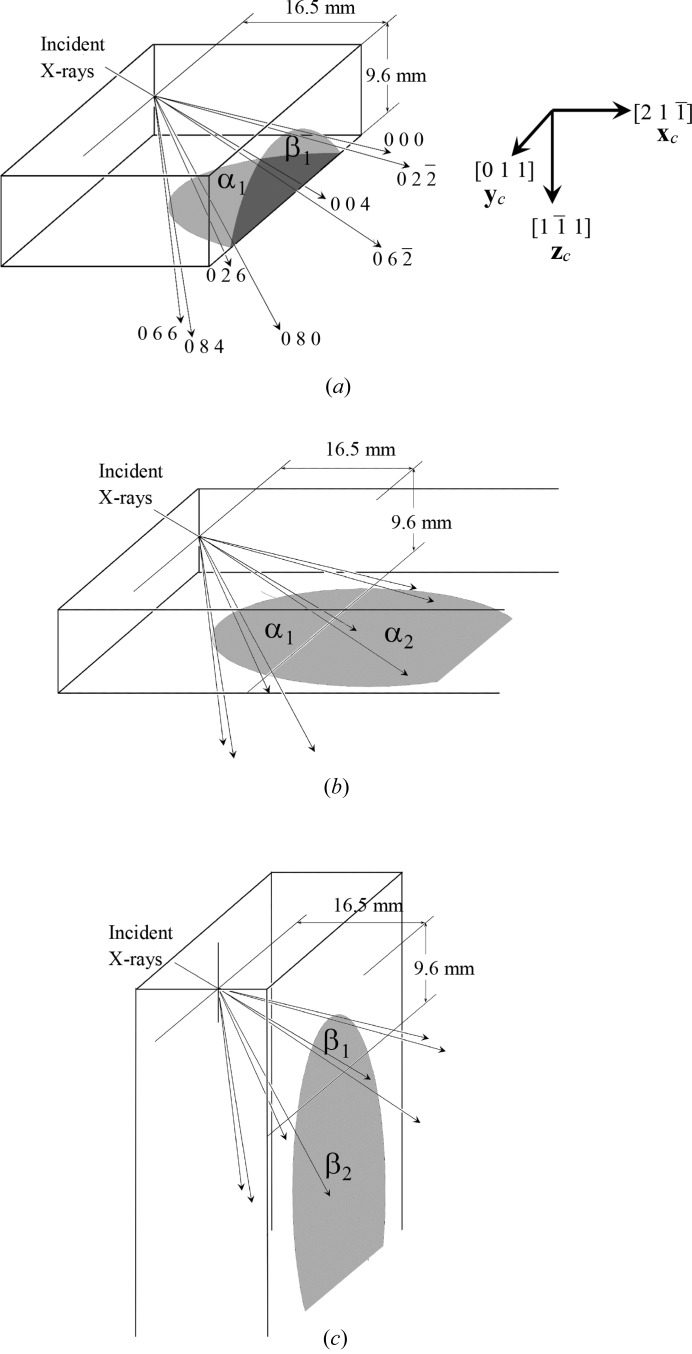
Geometry of the eight-beam pinhole topography. 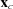, 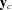 and 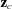 drawn in the upper-right corner are unit vectors in the directions 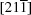, [011] and 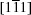, respectively.

**Figure 2:**
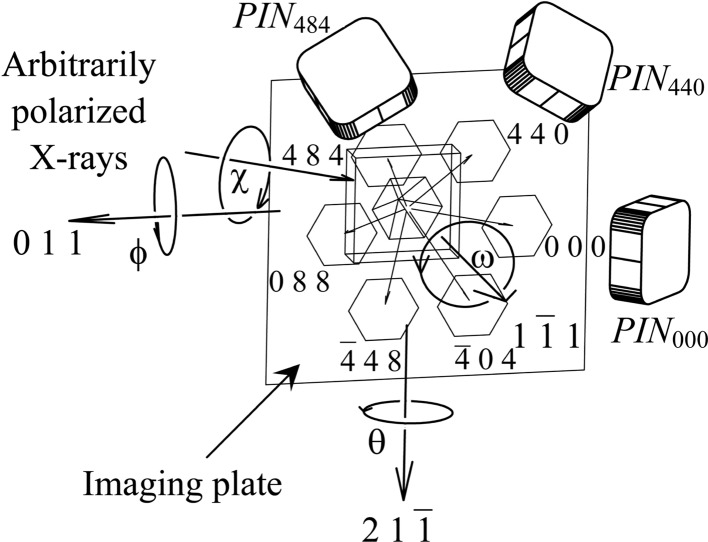
Reproduction of Fig. 7 in Okitsu *et al.* (2006 ▸), showing the six-beam pinhole topographs. An identical floating zone silicon crystal with a thickness of 9.6 mm was also used in the present experiment. However, the angles of the goniometers were adjusted such that the 000 forward-diffracted (FD) and 004, 026, 066, 084, 080, 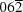 and 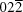 transmitted-reflected (TR) X-rays were simultaneously strong, as shown in Fig. 1 ▸(*a*).

**Figure 3:**
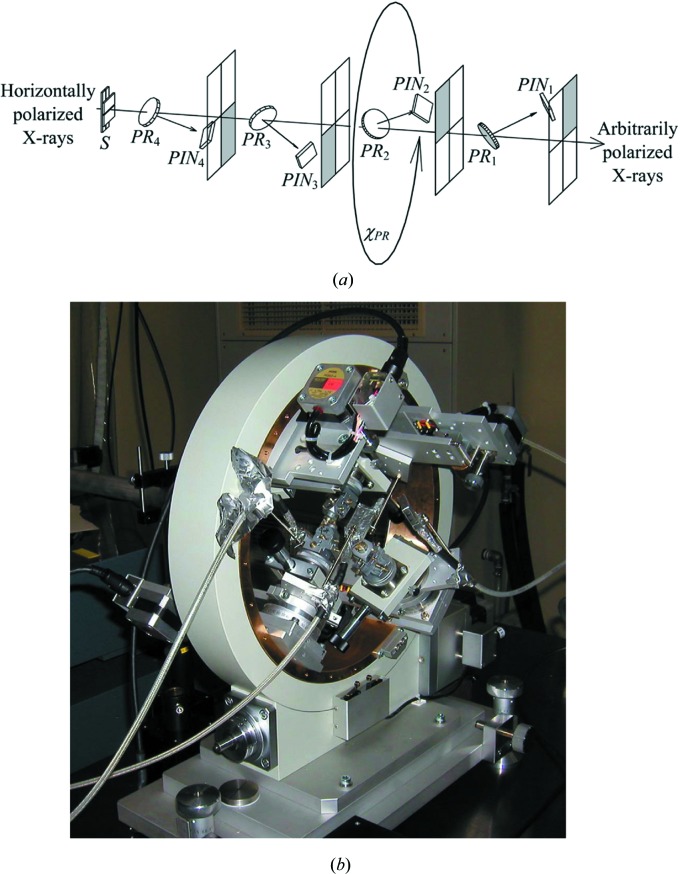
(*a*) Schematic drawing and (*b*) photograph of the rotating four-quadrant phase retarder system [reproduction of Fig. 3 of Okitsu *et al.* (2006 ▸)].

The optics used in the present work, shown in Fig. 1 ▸(*a*), were fundamentally the same as those in Fig. 2 ▸, which is a reproduction of Fig. 7 of O *et al.* 2006, showing the experimental setup used when taking the six-beam pinhole topographs. However, the goniometer axes were adjusted such that the 000 forward-diffracted (FD) and 004, 026, 066, 084, 080, 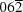 and 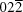 transmitted-reflected (TR) X-rays were simultaneously strong, and the vector product of the 000 FD and 066 TR beam directions was horizontal. The polarization state of the incident synchrotron X-rays with a photon energy of 18.245 keV at BL09XU of SPring-8 was controlled by using a rotating four-quadrant phase retarder system. Its schematic and photograph are shown in Figs. 3 ▸(*a*) and 3 ▸(*b*), respectively [these are reproductions of Figs. 3(*a*) and 3(*b*) of OIY 2012]. Its usage was described in O *et al.* 2006. An X-ray beam whose dimensions were limited to 25 × 25 µm was incident on a position on the entrance surface of a floating zone (FZ) silicon crystal with a thickness of 9.6 mm. The incident point was 16.5 mm from the corner of the crystal block, as shown in Fig. 1 ▸(*a*). The orientation of the crystal is also shown in Fig. 1 ▸. An imaging plate (IP) with a pixel size of 50 × 50 µm was placed 47.5 mm behind the crystal, such that its surface was approximately perpendicular to the [100] direction of the crystal.

## 3. Computer simulation   

### 3.1. Integration of the *n*-beam Takagi–Taupin equation   

**Figure 4:**
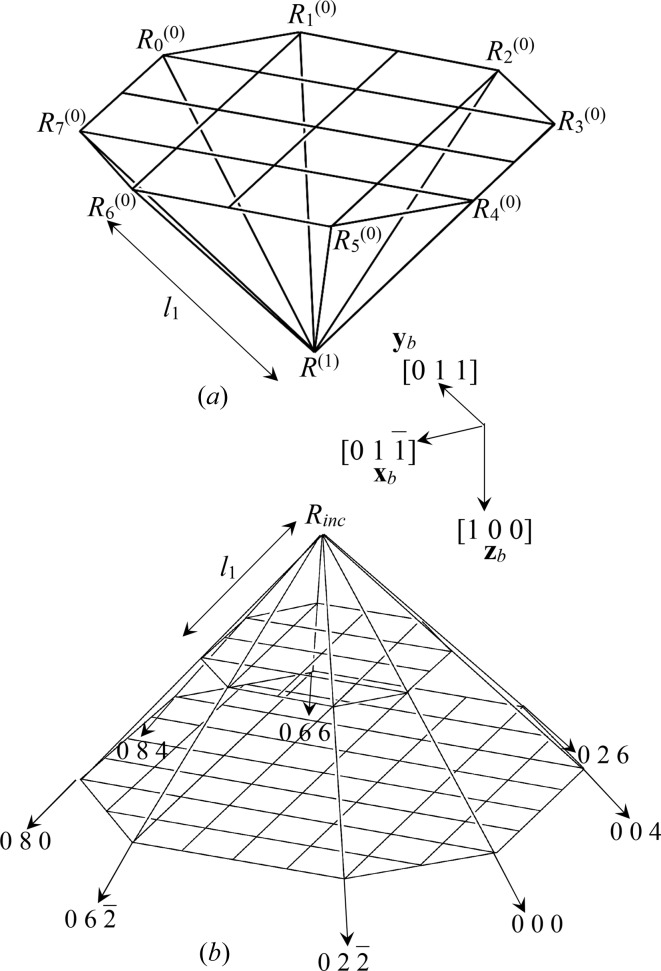
When integrating the *n*-beam T-T equation (performing the T-T simulation for *n* = 8), the crystal was divided into small octagonal pyramids. *l*_1_ was calculated to be 29.493 µm. *D*_*i*_^(*l*)^(*R* ^(1)^) can be calculated from *D*_*j*_^(*m*)^(*R*_*k*_^(0)^) (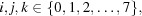 *l*, *m* ∈ {0, 1}) by solving equation (1).

The T-T simulations were performed in a similar manner to the approach described in O *et al.* 2006, except that the crystal was divided into small octagonal pyramids, as shown in Fig. 4 ▸(*b*). The calculated value of *l*_1_ in Fig. 4 ▸ was 29.493 µm. The height of the octagonal Borrmann pyramid was assumed to be 19.015 mm as calculated by 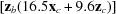
 mm, where 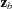, 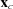 and 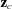 are unit vectors whose directions are as drawn in Figs. 1 ▸ and 4 ▸. The integration of the *n*-beam T-T equation was performed by solving equation (1) [see Fig. 4 ▸(*a*)] layer by layer whose thickness and normal direction were (19.015/4000) mm and [100], respectively: 
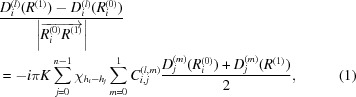
where *n* = 8. Equation (1) is a reproduction of equation (8) in O *et al.* 2006 with the lattice displacement term omitted. Here, *D*_*i*_^(*l*)^ and *D*_*j*_^(*m*)^ are the amplitudes of the *i*th-numbered *l*-polarized and *j*th-numbered *m*-polarized X-rays, where 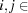 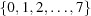 and *l*, *m* ∈ {0, 1}. 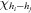 is the 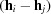
th-order Fourier coefficient of the electric susceptibility. *C*_*i*, *j*_^(*l*, *m*)^ is the polarization factor as defined later in equation (7). The T-T simulation took 15 h using one node of the supercomputer system ‘Sekirei’ of the Institute for Solid State Physics of the University of Tokyo. Each node (Intel Xeon E5-2680v3) had 24 cores. The program was then parallelized using Fortran 90 with *MPI* (Message Passing Interface).

As shown in Fig. 1 ▸(*a*), two vacuum parts are included in the Borrmann pyramid. Then, 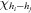 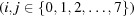 are assumed to be zero in the vacuum regions, as described in O *et al.* 2011. The reflection parameters as calculated using *XOP* 2.3 (Sanchez del Rio & Dejus, 1998 ▸) are used for the simulation, as summarized in Table 1 ▸.

### 3.2. Fast Fourier transformation of the rocking amplitude calculated using the Ewald–Laue theory   

The *n*-beam X-ray amplitude when the crystal is rotated two-dimensionally in the vicinity of the exact *n*-beam condition, with an incidence of plane-wave X-rays, can be obtained by solving the eigenvalue problem of the E-L theory.

In the T-T simulations reported by Okitsu and co-authors (O *et al.* 2006, O *et al.* 2011 and OIY 2012), a boundary condition that the incident X-rays have a nonzero amplitude only at the incident point on the crystal was assumed, *i.e.* the incident X-ray amplitude of the delta function was assumed as the boundary condition. The coincidence between the experimentally obtained and T-T simulated results implied that the physical properties of the pinhole topography could be approximated by the boundary condition of the delta function. Because a function of unity with an identical phase in reciprocal space is obtained by the Fourier transformation of the delta function in real space, the X-ray amplitudes of the *n*-beam pinhole topographs can also be obtained by Fourier transforming the X-ray amplitudes computed by solving the eigenvalue problem of the E-L theory.

E-L&FFT simulations of the *n*-beam section topography for *n* = 6 were reported by Kohn & Khikhlukha (2016 ▸) and Kohn (2017 ▸) for a symmetric six-beam Laue case. In this case, 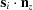 (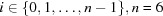) have identical values, where 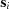 is a unit vector in the direction of the *i*th-beam propagation and 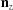 is a unit vector in the direction of the downward surface normal to the crystal. However, the eight-beam pinhole topography reported in the present work was performed for asymmetric Laue geometry, as shown in Fig. 1 ▸(*a*). Furthermore, the exit surface of the crystal was not a single plane. Accordingly, the procedure of the E-L&FFT simulation in the present work can be described as follows.

When plane-wave X-rays are incident on a parallel plate crystal, a Bloch wave 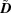 is excited as follows: 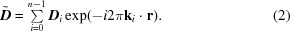
Here, *n* is the number of waves, 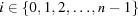, 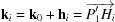, 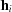 is the *i*th-numbered reflection vector, *P*_1_′ is the common initial point of the wavevector of the Bloch wave and 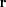 is the location vector. Laue’s fundamental equation of the dynamical theory (Laue, 1931 ▸; Authier, 2005 ▸) restricts the amplitude and wavevector of the Bloch wave as follows: 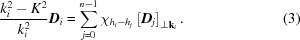
Here, *K* = 1/λ, where λ is the wavelength of the X-rays in vacuum and 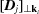 is the component vector of 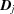 perpendicular to 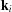. By applying the approximation *k*_*i*_ + *K* ≃ 2*K*, equation (3) becomes
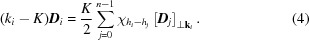
When 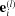 is the direction of polarization of the *i*th-numbered *l*-polarized X-ray beam, 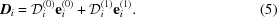


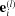
 was defined in the present work such that 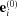 = 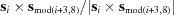 and 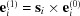. A form of the *n*-beam E-L theory that is applicable to asymmetric Laue geometry is found in Section 7.1.2 of Chang’s book (Chang, 2004 ▸). An algorithm to solve the eigenvalue problem was first developed by Colella (1974 ▸) and is more complex as it considers the second-order term. A simpler eigenvalue problem with a linear approximation found in Chang’s book can be described as follows: 
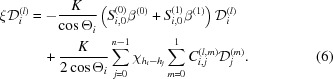
Here, the polarization factors *C* and *S* are defined as 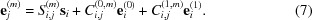
Equation (6) is the standard form of an eigenvalue problem, where 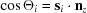. The values of Θ_*i*_ for Fig. 1 ▸(*b*) α and 1 ▸(*c*) β are summarized in Table 1 ▸. 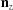 is the downward surface normal of the exit surface (but not the entrance surface as discussed later) of the crystal. The directions of 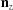 for Figs. 1 ▸(*b*) and 1 ▸(*c*) are 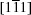 and 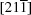, respectively, as shown in the upper-right corner of Fig. 1 ▸.

β^(0)^ and β^(1)^ are angular deviations of *P*_1_ from the Laue point, *La*, *i.e.* 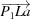 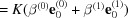. *P*_1_ is a point that exists on a spherical surface whose distance from *H*_0_ is *K*, where *H*_0_ is the origin of the reciprocal space. This spherical surface is assumed to be an approximate plane whose distance from *H*_0_ is *K* in the present work. The geometry around the Laue point is the same as that of Fig. 4 ▸ in Okitsu *et al.* (2019 ▸) (hereafter denoted OIY 2019). Equation (6) can be described using the matrix 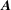 and vector 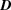 as follows: 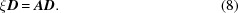
Here, 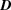 is a 2*n*-order column vector whose *q*th (*q* = 2*j* + *m* + 1) element is 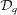, and 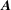 is a 2*n* × 2*n* matrix whose element of the *p*th (*p* = 2*i* + *l* + 1) row and *q*th column, 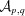, is as follows: 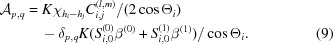
Here, δ_*p*,*q*_ is the Kronecker delta. When the X-ray amplitudes in α_1_ and α_2_, and those in β_1_ and β_2_, in Figs. 1 ▸(*b*) and 1 ▸(*c*), respectively, are calculated, Θ_*i*_^(α)^ and Θ_*i*_^(β)^ (summarized in Table 1 ▸) were substituted for Θ_*i*_ in equations (6) and (9). In general, equation (8) has 2*n* couples of eigenvalues ξ_*k*_ and eigenvectors 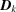 (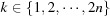). After obtaining these, the following equation is solved to satisfy the boundary condition that depends on the polarization state of the incident X-rays: 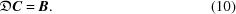
Here, 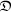 is a 2*n* × 2*n* matrix whose element of the *p*th row and *k*th column is 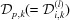, 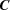 is a column vector whose *k*th element is 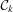, and 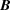 is a column vector of the boundary condition that depends on the polarization state of the incident X-rays. For 0- and 1-polarized incident X-rays, 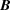 is 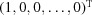 and 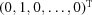, respectively.

**Figure 5:**
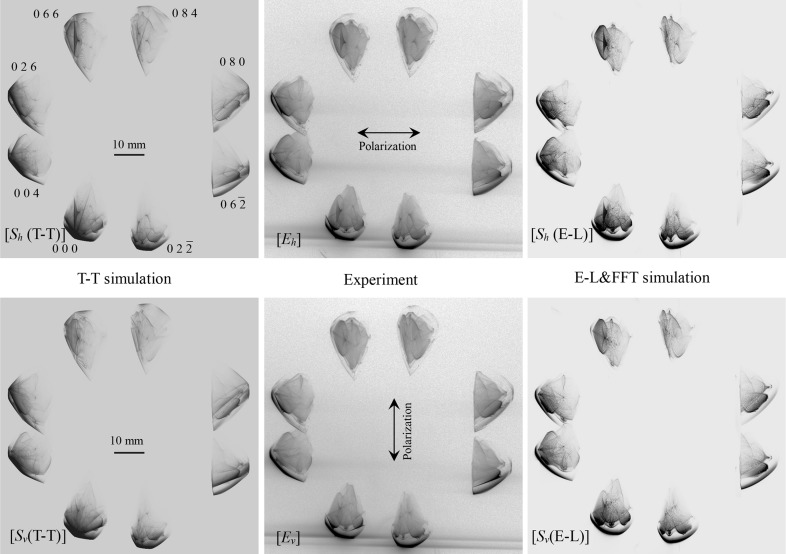
[*S*_*x*_(T-T)], [*E*_*x*_] and [*S*_*x*_(E-L)] (*x* ∈ {*h*, *v*}) are the T-T simulated, experimentally obtained and E-L&FFT simulated eight-beam pinhole topographs for horizontally (*x* = *h*) and vertically (*x* = *v*) polarized incident X-rays.

**Figure 6:**
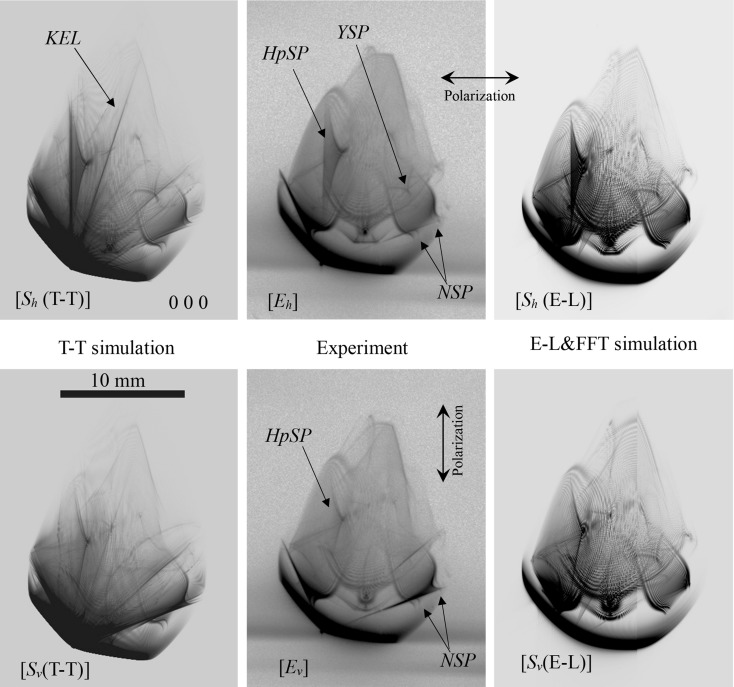
Enlargements of the 000 forward-diffracted images in Fig. 5 ▸.

**Figure 7:**
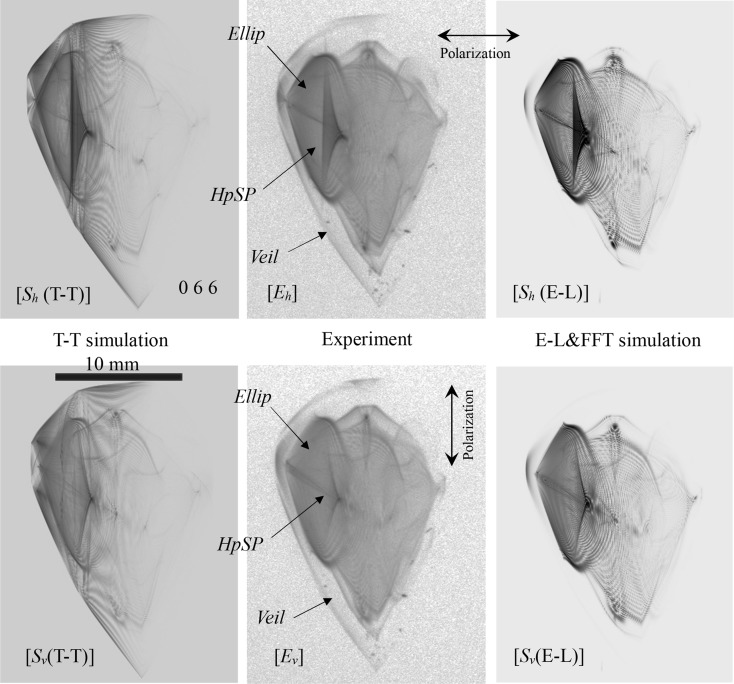
Enlargements of the 066 transmitted-reflected images in Fig. 5 ▸.

**Figure 8:**
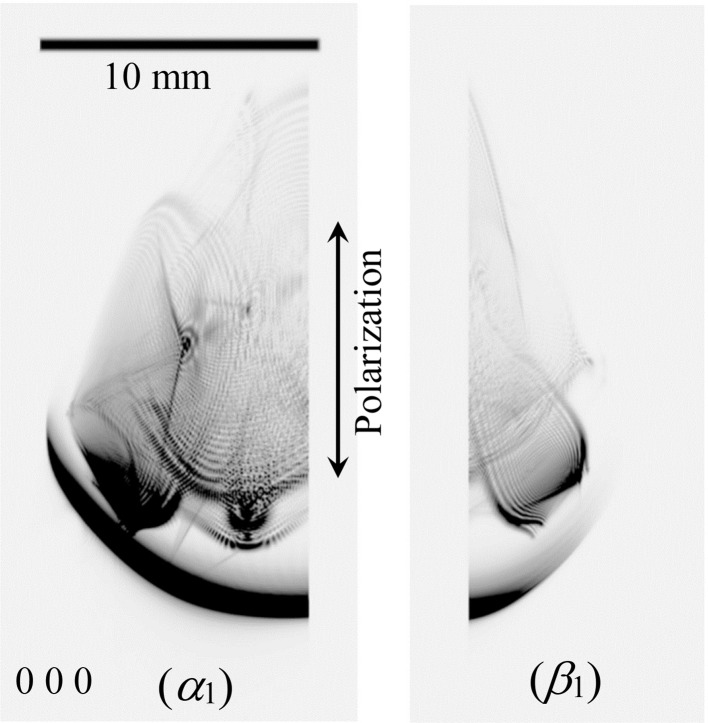
(α_1_) and (β_1_) are computed separately under the assumption of vertically polarized incident X-rays. These figures have been computed by projecting intensities of the 000 forward-diffracted X-rays on the exit planes α_1_ and β_1_ in Fig. 1 ▸(*a*) on the IP whose surface was normal to the [100] direction. X-ray intensities of α_2_ and β_2_ in Figs. 1 ▸(*b*) and 1 ▸(*c*) have been erased.

Incidentally, when *P*′_1,*k*_ is the common initial point of the *k*th Bloch wave, 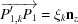. Here, let point *P*_1_′′ be defined such that 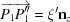 and 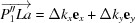, whereas 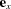 and 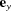 are unit vectors defined such that 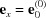 and 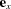, 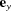 and 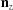 form a right-handed orthogonal system in this order (see Fig. 4 in OIY 2019). The total wavefield 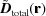 is excited by the incident plane-wave X-rays, and is given by 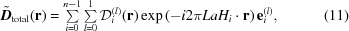
where 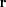 is a location vector in the crystal. The amplitude of the *i*th-numbered X-ray with polarization state *l* on the exit surface, 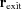, of the crystal, 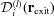, is given by
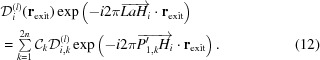
Here, 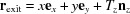 where *T*_*z*_ is the thickness of the crystal. Let the amplitude 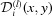 depend on two scalar values, *x* and *y*, then
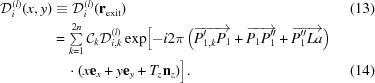
Since 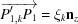, 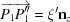 and 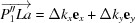 (see Fig. 4 in OIY 2019), then 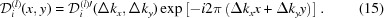
Here, 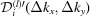 has been defined as follows: 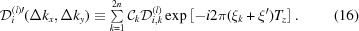
The wavefield *D*_*i*_^(*l*)^(*x*, *y*) excited by the incident X-rays whose wavefront is the delta function is a coherent superposition of the wavefield 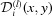 which is excited by the incident plane-wave X-rays with an amplitude of unity at the entrance surface of the crystal. Therefore, 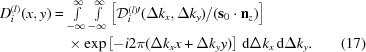
The term 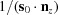 in the above equation is necessary to separately calculate the X-ray amplitudes of α_1_ and β_1_ in Fig. 1 ▸(*a*), as shown in Figs. 8(α_1_) and 8(β_1_) {from which Figs. 5 [*S*_*v*_(E-L)] and 6 [*S*_*v*_(E-L)] have been obtained}. The following assumptions are made: 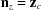 and *T*_*z*_ = 9.6 mm for Fig. 1 ▸(*b*); and 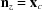 and *T*_*z*_ = 16.5 mm for Fig. 1 ▸(*c*). The term 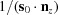 exists in equation (17) because the projection of the two-dimensional integration element over a plane normal to 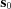 should be the same, even for different directions of 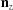. The calculated values of 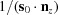 are 1.60518 and 1.74046 for Figs. 1 ▸(*b*) and 1 ▸(*c*), respectively. Since 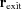 for 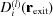 has a nonzero value only if it is inside the Borrmann pyramid, an infinitesimal spatial resolution is not necessary, and equation (17) can be replaced with a discrete Fourier transform as follows: 
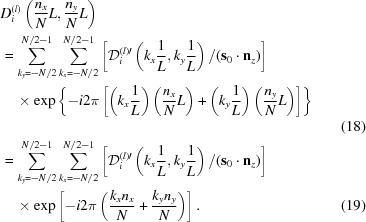
Here, *n*_*x*_, *n*_*y*_, *k*_*x*_ and *k*_*y*_ are integers, *N* is an even integer with which the lower and upper limits of the summations are determined, and *L* × *L* is the field size of a square that includes the topograph image. Therefore, Δ*k*_*x*_ = *k*_*x*_/*L*, Δ*k*_*y*_ = *k*_*y*_/*L*, *x* = *n*_*x*_ *L*/*N* and *y* = *n*_*y*_ *L*/*N*. If 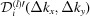 is a two-dimensional periodic function with a period 1/*L*, the contents in the summation of the right-hand side of equation (19) are evidently also a two-dimensional periodic function with a period *N*. Therefore, *D*_*i*_^(*l*)^(*n*_*x*_ *L*/*N*, *n*_*y*_ *L*/*N*) in the left-hand side of equation (18) is also a two-dimensional periodic function with a period of *L*. Then, equation (19) can be replaced by the following equation: 
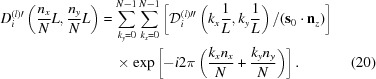
Equation (20) has a general form of a two-dimensional fast Fourier transform (FFT) (Cooley & Tukey, 1965 ▸). Therefore, *D*_*i*_^(*l*)^(*n*_*x*_ *L*/*N*, *n*_*y*_ *L*/*N*) can be obtained using an FFT as defined by equation (20). However, before performing the FFT, 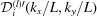 [− *N*/2 ≤ {*k*_*x*_, *k*_*y*_} ≤ *N*/2 − 1] in equation (19) should be replaced with 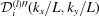 [0 ≤ {*k*_*x*_, *k*_*y*_} ≤ *N* − 1] in equation (20). Similarly after performing the FFT, *D*_*i*_^(*l*)^′(*n*_*x*_ *L*/*N*, *n*_*y*_ *L*/*N*) [0 ≤ {*n*_*x*_, *n*_*y*_} ≤ *N* − 1] in equation (20) should be replaced with *D*_*i*_^(*l*)^(*n*_*x*_ *L*/*N*, *n*_*y*_ *L*/*N*) [− *N*/2 ≤ {*n*_*x*_, *n*_*y*_} ≤ *N*/2 − 1] in equation (19). [*S*_*h*_(E-L)] and [*S*_*v*_(E-L)] shown in Figs. 5, 6, 7, 8(α_1_) and 8(β_1_) were obtained using the above procedure with *L* = 60 mm and *N* = 4096.

The eigenvalue/eigenvector problem described by equations (8) and (9) was solved using *ZGEEV* of *LAPACK*. The Fourier transform described by equation (20) was calculated with the FFT routine in the Intel *Math Kernel Library* (*MKL*). It took 470 s (280 s to solve the eigenvalue problem, 20 s for the FFT and 170 s to write the topographs to the hard disk) to obtain eight topograph images as shown in Fig. 5 ▸ [*S*_*h*_(E-L)] or [*S*_*v*_(E-L)] using one node (Intel Xeon E5-2680v3) of the supercomputer system ‘Sekirei’ of the Institute for Solid State Physics at the University of Tokyo.

## 4. Results   

Fig. 5 ▸ [*E*_*x*_] (*x* ∈ {*h*, *v*}) shows the experimentally obtained pinhole topograph images recorded on the IP for the incidence of the horizontally polarized (*x* = *h*) and vertically polarized (*x* = *v*) X-rays. Fig. 5 ▸ [*S*_*x*_(T-T)] and [*S*_*x*_(E-L)] show the T-T and E-L&FFT simulated images corresponding to Fig. 5 ▸ [*E*_*x*_]. In Figs. 6 ▸ and 7 ▸, enlargements of the 000 FD and 066 TR images, respectively, are shown. Fig. 6 ▸ [*E*_*h*_] and [*E*_*v*_] are reproductions of Fig. 11 [*S*(*a*)] and [*S*(*b*)] in OIY 2012. Fig. 6 ▸ [*S*_*h*_(T-T)] and [*S*_*v*_(T-T)] are obtained by solving the *n*-beam T-T equation layer by layer with a thickness of (19.015/4000) mm, whereas the number of layers was 3600 for Fig. 11 [*S*(*a*)] and [*S*(*b*)] in OIY 2012. In Figs. 6 ▸ and 7 ▸, there is good agreement between the experimentally obtained and computer-simulated topographs (both the T-T and E-L&FFT simulated ones).

A ‘harp-shaped’ pattern (*HpSP*), a pattern whose shape is similar to the alphabetical character ‘Y’ (*YSP*) and ‘nail-shaped’ patterns (*NSP*) in Fig. 6 ▸ [*E*_*h*_] are also shown in both Fig. 6 ▸ [*S*_*h*_(T-T)] and [*S*_*h*_(E-L)], revealing the equivalence between the T-T and E-L&FFT simulations. *NSPs* were also found in Fig. 6 ▸ [*S*_*v*_(T-T)], [*E*_*v*_] and [*S*_*v*_(E-L)]. The *HpSPs* in Fig. 6 ▸ [*S*_*v*_(T-T)], [*E*_*v*_] and [*S*_*v*_(E-L)] are practically the same but fainter than those in Fig. 6 ▸ [*E*_*h*_], [*S*_*h*_(T-T)] and [*S*_*h*_(E-L)], which shows an evident discrepancy owing to the polarization state of the incident X-rays. An *HpSP* is also found in an elliptical region (*Ellip*) in Fig. 7 ▸ [*E*_*h*_]. *HpSPs* and *Ellips* also exist in all of the experimentally obtained and computer-simulated images of Fig. 7 ▸. However, in Fig. 7 ▸, the *HpSP* for the incidence of vertically polarized X-rays is evidently fainter than that of the horizontally polarized X-rays.

In Fig. 7 ▸ [*E*_*x*_] and [*S*_*x*_(E-L)] (*x* ∈ {*h*, *v*}), the central region with a relatively strong X-ray intensity seems to be surrounded by a ‘veil’ with small X-ray intensities (*Veil*). However, such a faint region like a ‘veil’ is absent in the T-T simulated topographs.

As described in the last paragraph of Section 3, the calculation speed of the E-L&FFT simulation was over 100 times faster than the T-T simulation. The calculation speed of the E-L&FFT simulation is constant and independent of the crystal thickness, whereas that of the T-T simulation is proportional to the reciprocal of the thickness cubed. The superiority of this method reported by Kohn & Khikhlukha (2016 ▸) and Kohn (2017 ▸) was verified for a perfect crystal.

## 5. Discussion   

In Fig. 6 ▸ [*S*_*h*_(T-T)], a sharp line similar to a knife edge (*KEL*) is observed. However, a *KEL* is not seen in Fig. 6 ▸ [*E*_*h*_] or [*S*_*h*_(E-L)]. The width of the *KEL* is extremely narrow. In the case of the T-T simulation, a boundary condition of the incident X-rays whose amplitude is the delta function was assumed. Then the incident X-rays have a plane-wave component whose initial point of the wavevector was far from the Laue point. However, the incident X-rays used in the experiment have a finite angular width. In addition, in the E-L&FFT simulation, the integration range is finite. This is probably the reason for the absence of the *KEL* in Fig. 6 ▸ [*E*_*h*_] and [*S*_*h*_(E-L)].

With regard to the *Veil*, this pattern may be explained by the following hypothesis. When the crystal is thick in the case of two-beam section topography in general, dark areas are observed in the forward-diffracted and transmitted-reflected topographs on both sides of the central bright region due to the Borrmann effect. The *Veil* may correspond to these dark areas excited by the incident X-ray plane-wave component whose initial point of the wavevector is distant from the Laue point. The *Veil* can be observed in all *h*_*i*_ *k*_*i*_ *l*_*i*_-diffracted images (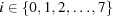) in Fig. 5 ▸ [*E*_*x*_] and [*S*_*x*_(E-L)] (*x* ∈ {*h*, *v*}). However, it cannot be found in Fig. 5 ▸ [*S*_*x*_(T-T)] (*x* ∈ {*h*, *v*}). This feature of the *Veil* may be explained by the weak or zero intensity of the X-ray plane-wave component whose incident angle is far from the exact eight-beam condition.

When the rocking curves of the X-rays are discussed in the E-L theory in general, the downward surface normal is assumed to be perpendicular to the entrance surface of the crystal. Its directions for Figs. 1 ▸(*b*) and 1 ▸(*c*) are perpendicular to each other. However, Figs. 8 ▸(α_1_) and 8 ▸(β_1_) are smoothly linked as shown in Fig. 6 ▸ [*S*_*v*_(E-L)]. When performing the E-L&FFT simulation, the direction of the entrance surface of the crystal does not have to be considered, whereas the direction of the exit surface was important. Thus, the X-ray intensity on the exit surface of the crystal does not depend on the shape of the entrance side of the crystal.

The X-ray *n*-beam rocking amplitude from a planar perfect crystal can also be obtained by solving the *n*-beam T-T equation, which will be reported in the near future. Since the result of the E-L&FFT simulation is verified to be independent of the shape of the entrance surface, the same result as reported in the present work should be obtained by the fast Fourier transform of the rocking amplitude calculated by the T-T equation.

## 6. Summary   

Experimentally obtained and computer-simulated (both T-T simulated and E-L&FFT simulated) asymmetric eight-beam pinhole topographs, which were in good agreement, were reported. This is for a case where the exit surface was not a single plane. It was verified that the X-ray wavefield could be computed not only based on the *n*-beam T-T equation but also on the *n*-beam E-L theory.

**Figure 9:**
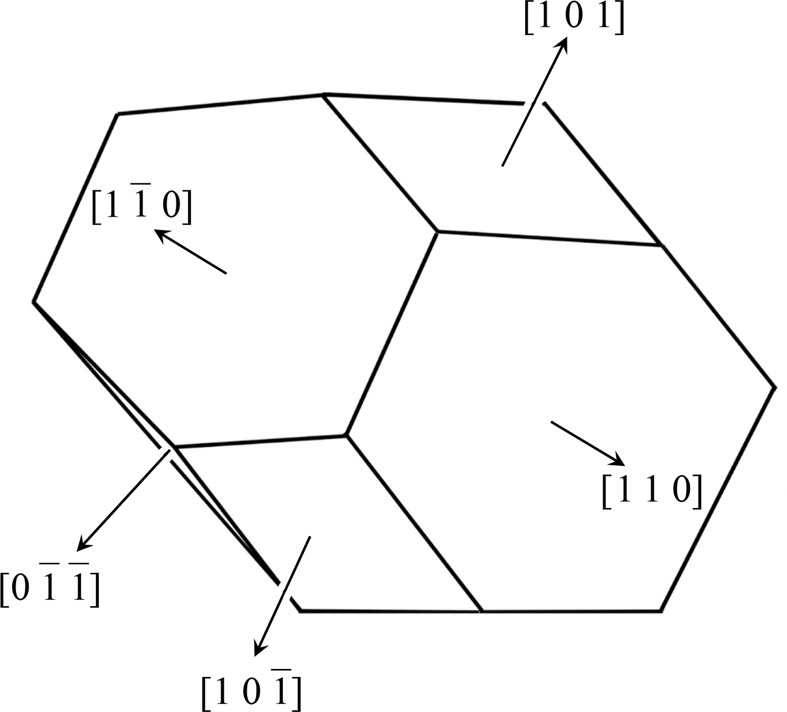
Outline drawing of a tetragonal lysozyme crystal with 12 facets.

The present work has provided the first demonstration of the E-L&FFT simulation overcoming difficulties when calculating the X-ray intensities diffracted from such a complex-shaped crystal as shown in Fig. 9 ▸ to verify the first hypothesis concerning an excessively large *R* factor in a protein crystal structure analysis.

## Figures and Tables

**Table 1:** 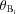
 is the Bragg angle, Θ_*i*_^(α)^ is an angle spanned by 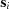 and 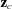, Θ_*i*_^(β)^ is that spanned by 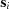 and 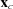, calculated for the geometries of Figs. 1 ▸(*b*) and 1 ▸(*c*), respectively; 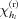 and 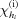 are the real and imaginary parts, respectively, of 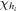, which is the 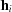
th-order Fourier coefficient of the electric susceptibility as calculated using *XOP* 2.3 (Sanchez del Rio & Dejus, 1998 ▸)

Ordinal number *i*	*h_i_*	*k_i_*	*l_i_*	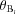 (°)	Θ_*i*_^(α)^ (°)	Θ_*i*_^(β)^ (°)	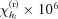	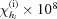
0	0	0	0	0.0000	51.4657	54.9312	−2.914850	−1.333430
1	0	0	4	14.4925	24.2271	68.2692	+1.444290	+1.251020
2	0	2	6	23.3087	24.2271	68.2692	−1.006410	−1.136870
3	0	6	6	32.0640	51.4657	54.9312	+0.617250	+1.000700
4	0	8	4	34.0269	70.4859	38.8424	+0.545491	+0.969283
5	0	8	0	30.0335	87.4153	10.5211	−0.699980	−1.033130
6	0	6	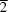	23.3087	87.4153	10.5211	+1.006410	+1.136870
7	0	2	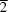	10.1925	70.4859	38.8424	−1.766730	−1.291570
